# Siglec-15 Promotes Evasion of Adaptive Immunity in B-cell Acute Lymphoblastic Leukemia

**DOI:** 10.1158/2767-9764.CRC-23-0056

**Published:** 2023-07-17

**Authors:** Claire E. Pillsbury, Jodi Dougan, Jennifer L. Rabe, Jairo A. Fonseca, Chengjing Zhou, Alyssa N. Evans, Hasan Abukharma, Ona Ichoku, Gloria Gonzalez-Flamenco, Sunita I. Park, Ahmed Aljudi, Deborah DeRyckere, Sharon M. Castellino, Sarwish Rafiq, Solomon Langermann, Linda N. Liu, Curtis J. Henry, Christopher C. Porter

**Affiliations:** 1 Cancer Biology Program, Laney Graduate School, Emory University, Atlanta, Georgia.; 2 Department of Pediatrics, Emory University School of Medicine, Atlanta, Georgia.; 3 Molecular Biology Program, University of Colorado Denver, Aurora, Colorado.; 4 Winship Cancer Institute, Emory University, Atlanta, Georgia.; 5 NextCure, Inc. Beltsville, Maryland.; 6 Clinical Laboratory, Children's Healthcare of Atlanta, Atlanta, Georgia.; 7 Department of Pathology, Emory University School of Medicine, Atlanta, Georgia.; 8 Aflac Cancer and Blood Disorders Center, Children's Healthcare of Atlanta, Atlanta, Georgia.

## Abstract

Siglec-15 (Sig15) has been implicated as an immune checkpoint expressed in solid tumor-infiltrating macrophages and is being targeted in clinical trials with mAbs to normalize the tumor immune microenvironment and stimulate antitumor immunity. However, the role of Sig15 in hematologic malignancies remains undefined. Sig15 mRNA and protein expression levels in hematologic malignancies were determined from publicly available databases, cell lines, and primary patient samples. Human B-cell acute lymphoblastic leukemia (B-ALL) cell lines were used to identify signaling pathways involved in the regulation of Sig15 expression. Secreted/soluble Sig15 and cytokine levels were measured from the plasma of children with leukemia and healthy controls. Knockdown and knockout of *Siglec15* in a murine model of B-ALL was used to evaluate the effect of leukemia-derived Sig15 on the immune response to leukemia. We observed pathologic overexpression of Sig15 in a variety of hematologic malignancies, including primary B-ALL samples. This overexpression was driven by NFκB activation, which also increased the surface localization of Sig15. Secreted/soluble Sig15 was found to circulate at elevated levels in the plasma of children with B-ALL and correlated with an immune-suppressive cytokine milieu. Genetic inhibition of Sig15 in murine B-ALL promoted clearance of the leukemia by the immune system and a marked reversal of the immune-privileged leukemia bone marrow niche, including expanded early effector CD8^+^ T cells and reduction of immunosuppressive cytokines. Thus, Sig15 is a novel, potent immunosuppressive molecule active in leukemia that may be targeted therapeutically to activate T lymphocytes against leukemia cells.

**Significance:**

We demonstrate that Sig15 is overexpressed in hematologic malignancies driven by NFκB, is required for immune evasion in a mouse model of leukemia, and, for the first time, that it circulates at high levels in the plasma of children with leukemia.

## Introduction

B-cell acute lymphoblastic leukemia (B-ALL) is the most frequent subtype of acute lymphoblastic leukemia and is the most common leukemia in children (1). Though standard-of-care treatment for ALL is sufficient to induce complete remission in up to 98% of cases (2), relapse occurs in approximately 15%–20% of cases (3) and is characterized by highly aggressive disease, resistance to most salvage therapies, and historic 5-year survival rates of below 50% (4). Treatment of refractory B-cell malignancies using CD19-targeted chimeric antigen receptor (CAR) expressing T cells and blinatumomab, a CD19-CD3 bispecific antibody, have shown dramatic potential (5, 6), highlighting the potential of harnessing the immune system to treat these diseases. Nonetheless, responses are often incomplete or short lived, and there remains much to understand about the mechanisms of immune evasion critical to disease progression and the best strategies to fully optimize therapeutic immunomodulation for B-cell malignancies.

In previous work, we noted differential expression of *Siglec15* (Sig15) in an immunogenic mouse model of B-ALL as compared with the non-immunogenic control leukemia (7). Sig15 is a member of the sialic acid binding Ig-like lectin (Siglec) family of proteins, which are known to bind sialic acid sugar moieties on a variety of proteins and have immune stimulatory or suppressive signaling effects in select immune subsets (8). These effects are mediated by either the presence of immunoreceptor tyrosine-based inhibition motif domains or positively-charged amino acid residues located within their transmembrane domains; the latter allows for coupling and stabilization of the immunoreceptor tyrosine-based activation motif (ITAM) domains of partner adaptor proteins to enable downstream signaling activation (9).

Sig15 is known to be expressed in certain subsets of dendritic cells and macrophages (10, 11), as well as mature osteoclasts (12, 13), where it has been demonstrated to be critical for osteoclast differentiation and physiologic bone growth/remodeling (14, 15). It possesses a lysine residue in its transmembrane domain which allows it to couple with the ITAM domains of adapter proteins DAP12 and FcRγ to activate downstream Syk and ERK/Akt in osteoclasts and myeloid cells (10), a pathway that can be exploited by tumor cells to promote TGFβ secretion in adjacent tumor-associated macrophages (16). A seminal study of Sig15 in non–small cell lung cancer (NSCLC), primarily as expressed by tumor-infiltrating myeloid cells, suggests a role for this molecule in suppressing antigen-specific T-cell activation (11). Further studies in clear-cell renal cell carcinoma and osteosarcoma have implicated an immunosuppressive function for this molecule across solid tumor types (17, 18). However, the unique regulation and underlying mechanisms of immunomodulation by Sig15, particularly in leukemias and other hematologic malignancies, remains to be defined.

Our investigations detected high Sig15 expression across hematologic malignancies in both immortalized cell lines and primary patient samples. In the context of B-ALL, we have characterized a novel intracellular localization pattern and regulatory axis for Sig15. We have also demonstrated for the first time the release of a secreted or soluble form of Sig15 which circulates in the plasma of pediatric patients with B-ALL and correlates with markers of systemic immunosuppression. Silencing of Sig15 expression in a murine model of B-ALL results in higher expansion and activation of select T-cell populations and a decrease in immunosuppressive cytokines in the bone marrow, which suggests a niche for Sig15 as a target for therapeutic development in hematologic malignancies.

## Materials and Methods

### Cell Lines and Tissue Culture

The human B-ALL cell lines REH (RRID:CVCL_1650), RCH-ACV (RRID:CVCL_1851), SEM RRID:CVCL_0095), Nalm6 (RRID:CVCL_0092), KOPN8 (RRID:CVCL_1866), RS411 (RRID:CVCL_0093), and 697 (RRID:CVCL_0079) were obtained from ATCC and grown in RPMI1640 or Iscove's Modified Dulbecco's Medium media supplemented with either 10% or 20% FBS and 1% penicillin/streptomycin. Human cell lines were not authenticated, but were periodically tested for *Mycoplasma* by RT-PCR (most recently in June, 2022). The luciferase-expressing, p185 *BCR-ABL1^+^ Arf^−^^/^^−^* murine B-ALL cell line was generously provided by Dr. Richard Williams (St. Jude Children's Research Hospital, Memphis, TN; refs. 19–21). This cell line was cultured in RPMI1640 supplemented with 20% FBS, 1% penicillin/streptomycin, and 0.1% β-Mercaptoethanol, and was periodically tested for *Mycoplasma* by RT-PCR (most recently in November, 2022). All cell lines were cultured in a 37°C humidified incubator with 5% CO_2_ for a maximum of 2 months before thawing a new vial of frozen stock.

To knockout *Siglec15*, the murine leukemia cells were transduced with a pCW-Cas9 construct (RRID:Addgene_50661) for doxycycline-inducible, FLAG-tagged Cas9 expression, as well as two guide RNA (gRNA) sequences (Supplementary Table S1) cloned into the pLX-sgRNA (RRID:Addgene_50662) plasmid. Cas9 expression was induced with 10 μg/mL blasticidin for 3 days and cells were then seeded for single-cell isolation. Monoclonal colonies were harvested and assessed for genetic knockout using qRT-PCR. Short hairpin RNA (shRNA) targeting *Siglec15* expressed from pLKO.1 (shSig15; Sigma-Aldrich; TRCN0000255303) was used to knock down expression with a non-silencing sequence as a control (shNS; Sigma-Aldrich, SHC002).

CAR expressing Jurkat cells were generated by retrovirally transducing Jurkats (RRID:CVCL_0065) with a CAR containing a CD19-binding domain, the CD28 transmembrane and signaling domain and the CD3ζ signaling domain, as described previously (22). CAR-expressing Jurkats and CD19^+^ REH cells were cocultured at a 2:1 effector:target ratio, at which point 50 μg/mL of human IgG1 (Abcam) or His-tagged recombinant SIGLEC15 protein (ABclonal) was added to the coculture and incubated for 4 hours prior to analysis.

### Quantitative Gene and Protein Analysis

For gene expression analysis, human cell lines were harvested at a concentration of 8 × 10^5^ cells/mL, and RNA was isolated from these samples using the Quick-RNA Miniprep Kit (Zymo Research). RNA was reverse transcribed using the High-Capacity cDNA Reverse Transcription Kit (Thermo Fisher Scientific) and run in technical triplicate for qRT-PCR reactions utilizing the SYBR Green PCR Master Mix (Thermo Fisher Scientific). Sig15 expression was normalized using reference genes GAPDH and 18srRNA. Publicly available data were queried from the Oncomine (https://www.oncomine.org; accessed online May 2018), TARGET ALL Phase 2 (https://ocg.cancer.gov/programs/target; accessed online October 2020), and St. Jude PeCan (https://pecan.stjude.cloud/; accessed online August 2020) databases. For survival analyses relative to mRNA expression, the median *SIGLEC15* level was used as a cutoff to define high versus low expression.

For protein expression analysis, human cell lines were harvested at a concentration of 8 × 10^5^ cells/mL and lysed on ice for 45 minutes in RIPA lysis buffer (Thermo Fisher Scientific) containing protease inhibitors (Roche). Lysates were then clarified at 14,000 × *g* for 10 minutes and quantified using the BCA protein assay kit (Thermo Fisher Scientific). Western immunoblots were probed for Sig15 using a polyclonal antibody from Invitrogen (1:2,000) and mAbs from Creative Biolabs (Clone A9E8; 1:1,000) and NextCure (Clone 1F7; 2 μg/mL). Additional probes included DAP12 (Sigma-Aldrich; 1:250), FcRγ (Cell Signaling Technology; 1:1,000), and B-actin (Sigma-Aldrich; Clone AC-15; 1:2,000).

### Immunofluorescence

Human B-ALL cells were seeded at a concentration 5 × 10^5^ cells/mL to a poly-L-lysine (Sigma-Aldrich)-coated 8-well removable chamber microscope slide (ibidi) and allowed to adhere for 24 hours at 37°C prior to treatment. At harvest, the wells were washed twice with cold PBS, fixed with 4.2% paraformaldehyde (PMA) at 4°C for 10 minutes, permeabilized with 0.2% Triton X-100 (Sigma Aldrich) for 10 minutes at room temperature, and blocked for 30 minutes at room temperature. Wells were stained for Sig15 (Invitrogen; 1:400) and Golgin-97 (Thermo Fisher Scientific; Clone CDF4; 1:200) for 20 minutes at room temperature, washed in triplicate with PBS, and stained with fluorescent secondary antibodies against either antigen. Slides were mounted using Prolong Gold Antifade with DAPI (Invitrogen) and imaged on an Olympus FV1000 confocal microscope. All colocalization analysis was performed using the Coloc2 plugin in Fiji (23).

### Primary Human Samples

All research involving samples from human subjects was approved by the Institutional Review Board (IRB) of Emory University (Atlanta, GA) and performed in accordance with recognized ethical guidelines (e.g., U.S. Common Rule). Plasma samples from pediatric patients with leukemia were obtained from the Leukemia/Lymphoma Biorepository of the Aflac Cancer and Blood Disorders Center at Children's Healthcare of Atlanta (IRB#34535). Peripheral blood and plasma from healthy individuals were obtained from the Clinical and Translational Discovery Core at Emory University (Atlanta, GA; IRB#89506). All samples were acquired after written informed consent was provided. Residual, fresh bone marrow aspirate and peripheral blood samples from pediatric patients with B-ALL were analyzed for Sig15 expression by flow cytometry under protocol (IRB#96145). Bone marrow aspirate from healthy donors was purchased from AllCells.

### Flow Cytometry

Human cell lines were harvested at a concentration of 8 × 10^5^ cells/mL and stained with LIVE/DEAD Fixable Aqua Stain (Thermo Fisher Scientific) and Sig15 (NextCure; Clone NP159; 1:100), washed with PBS in triplicate, and stained with an Alexa Fluor 488 goat anti-mouse secondary antibody. Intracellular stains were performed using the BD Cytofix/Cytoperm (BD Biosciences) kit. Fresh pediatric B-ALL samples from bone marrow aspirate (BMA) and peripheral blood lymphocytes (PBL) were collected, counted, washed, and then stained with a preselected antibody cocktail (Supplementary Table S2). Samples were then RBC-lysed with Ammonium Chloride Lyse Reagent (Medialab), washed, decanted, and fixed with 1% buffered formalin (Medialab) prior to flow cytometric analysis. FlowJo Software (BD Biosciences; RRID:SCR_008520) was used for all flow cytometry data analyses.

### Secreted/Soluble Sig15 and Cytokine/Chemokine Detection

Aliquots from plasma samples and human B-ALL cell line supernatants were analyzed for Sig15 using electrochemiluminescence on a Meso QuickPlex reader (Meso Scale Diagnostics). Patient plasma was also analyzed for cytokines and chemokines using the Cytokine 35-plex Human Panel (Thermo Fisher Scientific) for the Luminex 200 System, which has been reported previously (24). Secreted or soluble Sig15 (sSig15) was captured using the 5G12 mAb (NextCure) and detected via the NP159 antibody (NextCure) for analysis. A subset of reliably detected cytokines was selected for correlation with sSig15 levels. Data were log_2_ transformed prior to analyses using Pearson correlation test. Pearson *r* values are depicted in the heat map that was generated using Morpheus (www.software.broadinstitute.org/Morpheus).

### *In Vivo* Experiments

C57BL/6 mice of either wildtype (WT; RRID:MGI:2159769) or *Rag1^−^^/^^−^* (B6.129S7-Rag1^tm1Mom^/J; RRID:IMSR_JAX:002216) background were obtained from the Jackson Laboratory. Female mice used in this study were 4–8 weeks of age. Mice were housed in pathogen-free conditions in the Division of Animal Resources Facility in the Health Sciences Research Building on Emory University campus (Atlanta, GA). All animal studies in this investigation were approved by the Emory University Institutional Animal Care and Use Committee (Atlanta, GA).

For survival experiments, unirradiated WT or *Rag1^−^^/^^−^* mice were injected with 2 × 10^5^ murine B-ALL cells via tail vein injection. Isoflurane-anesthetized mice were intraperitoneally injected with luciferin and imaged on the *In Vivo* Image System (IVIS) Spectrum (Perkin Elmer; RRID:SCR_020397) to measure leukemia progression. Recipient mice were removed from the study upon either manifesting an ill appearance or when their luciferase signal exceeded 10^8^ photons/second based on previous experience (7).

For short-term immunophenotyping experiments, WT mice were injected with 2 × 10^5^ leukemia cells and monitored for 7 days, at which point all recipients were euthanized. Bone marrow supernatant was harvested and analyzed using the Mouse Cytokine 44-plex Array (Eve Technologies). Bone marrow cells were harvested and analyzed via flow cytometry using a multiparameter panel on a Cytek Aurora (RRID:SCR_019826; Supplementary Table S2).

### Statistical Analyses

Statistical analyses were performed using GraphPad Prism software (RRID:SCR_002798). Statistical significance between groups was determined using ANOVA with Tukey multiple comparisons test, unless otherwise specified. Error bars in figures represent the SD and may be obscured when narrow. Animal experiments included at least 3 mice per group and were repeated at least once. Data from all mice are included in the results.

### Data Availability Statement

The results published here include data generated by the Therapeutically Applicable Research to Generate Effective Treatments (https://ocg.cancer.gov/programs/target) initiative, phs000218. The data used for this analysis are available at https://portal.gdc.cancer.gov/projects.

## Results

### Sig15 is Widely Expressed Across Hematologic Malignancies

**FIGURE 1:**
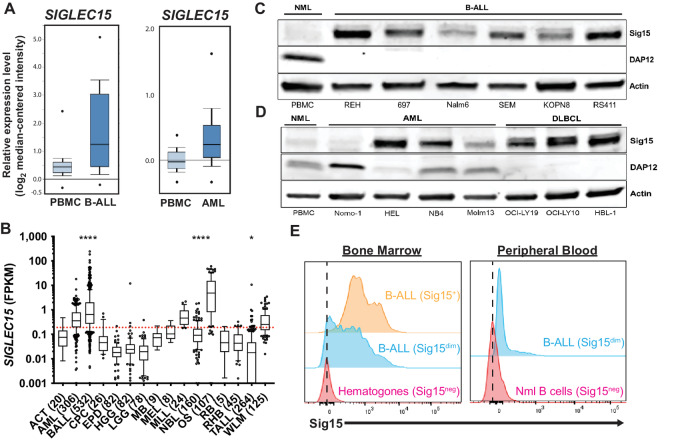
Sig15 is highly expressed in B-ALL and additional hematologic malignancies. **A,** Relative *SIG15* expression. B-ALL (*N* = 147; *P* = 3.6 × 10^−19^) and AML (*N* = 542; *P* = 2.0 × 10^−9^) samples showed higher *SIG15* than normal PBMC samples (*N* = 74). Data adapted from Haferlach and colleagues through Oncomine.org (53). **B,** Relative *SIG15* expression across a panel of common childhood cancers from the St. Jude PeCan database. Dotted red line indicates median expression for all tumors in the graph (*, *P* < 0.05; ****, *P* < 0.0001, ANOVA). Western blot analysis shows higher SIG15 expression across a panel of B-ALL (**C**), AML (**D**), and DLBCL human cell lines compared with normal healthy PBMCs (NML). No B-ALL or DLBCL cell lines show detectable levels of the Sig15 binding partner DAP12. SIG15 was probed using the Invitrogen polyclonal antibody. **E,** Representative flow cytometry of primary childhood B-ALL. Three of seven BMA samples had B-ALL cells which stained positive/dim-positive for Sig15 and one of eight PBL samples had B-ALL cells which stained positive. Zero of two hematogones from non-leukemia donor BMA samples and zero of six B cells from PBL samples from non-leukemia donors were positive for Sig15. Dotted lines represent the mean fluorescence intensity from normal hematogones and B cells. SIG15 was probed using the NP159 mAb (NextCure) conjugated to Alexa Fluor 647.

Sig15 expression has been documented in subsets of dendritic cells, macrophages, and mature osteoclasts, and there exists some evidence that it is also expressed in some acute myeloid leukemias (AML; refs. 25, 26). The extent of Sig15 expression in hematologic malignancies, specifically of lymphoid origin, remains undefined. We queried the Oncomine cancer microarray database, finding higher *SIGLEC15* expression in both B-ALL and AML patient samples compared with healthy donor peripheral blood mononuclear cells (PBMC; Fig. 1A). From the St. Jude PeCan database, among common pediatric cancers, we found higher *SIGLEC15* expression in B-ALL, AML, mixed lineage leukemia, and osteosarcoma relative to the median expression across all tumors (Fig. 1B). Among pediatric B-ALL subtypes, *SIGLEC15* expression was highest in patients with the *ETV6-RUNX1* translocation (Supplementary Fig. S1A), the most common fusion gene in childhood ALL (27). Notably, higher expression of *SIGLEC15* mRNA in the bone marrow was associated with longer event-free survival in a cohort of 98 children with B-ALL enriched for those with early relapse (Supplementary Fig. S1B).

Across a panel of eight immortalized human B-ALL cell lines, we observed higher SIGLEC15 expression compared with healthy donor PBMCs (Fig. 1C) by Western blot analysis. This was consistent across AML, diffuse large B-cell lymphoma (DLBCL), and T cell ALL (T-ALL) cell lines, as well as a single chronic myelogenous leukemia cell line (Fig. 1D; Supplementary Fig. S1B). We also performed flow cytometry for Sig15 on primary B-ALL cells gated from fresh BMA and PBL samples from pediatric patients with B-ALL. Three of seven pediatric B-ALL BMA and one of eight PBL samples stained positive/dim-positive for Sig15 on their surface, while all normal bone marrow hematogones and circulating B cells from non-leukemia donors (*n* = 8) were Sig15-negative (Fig. 1E).

### The Expression and Localization of Sig15 are Regulated by NFκB

**FIGURE 2:**
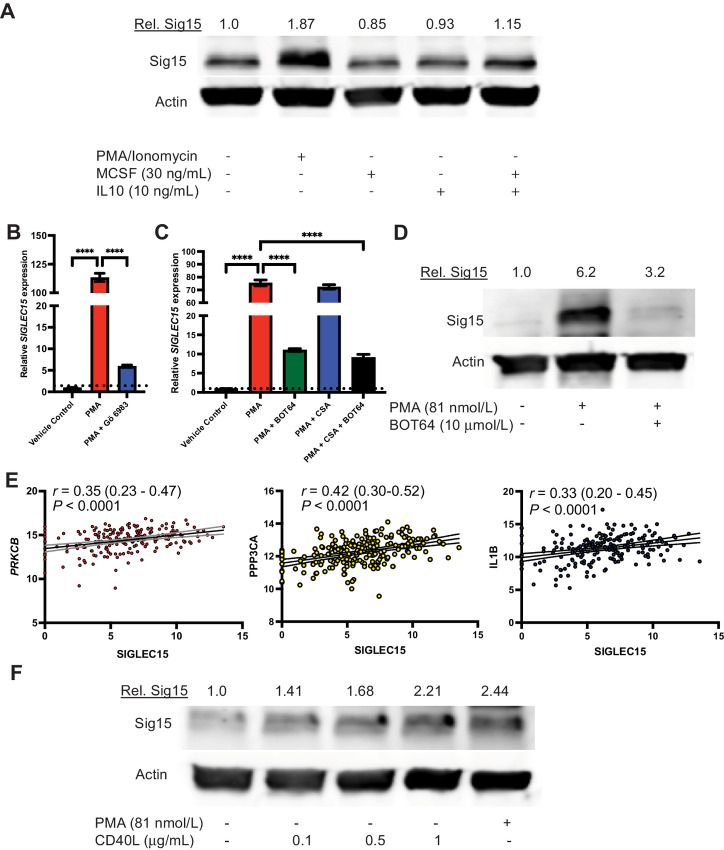
Sig15 expression is regulated by NFκB activation in B-ALL. **A,** Western blot analysis shows upregulated SIG15 expression in REH cells stimulated for 24 hours with a PMA/ionomycin stimulation (81 nmol/L PMA and 1.3 μmol/L ionomycin) but not with 30 ng/mL of recombinant human MCSF and/or IL10. Densitometric analysis of SIG15 is quantified above. SIG15 was probed using the Invitrogen polyclonal antibody. **B,** qRT-PCR from REH cells treated with PMA (81 nmol/L) with or without pan-PKC inhibitor Gö 6983 (5 μmol/L) for 24 hours, showing that PKC inhibition abrogates *SIG15* induction by PMA. (****, *P* < 0.0001). **C,** qRT-PCR from REH cells stimulated with PMA (81 nmol/L) and/or treated with calcineurin inhibitor CSA (1 μmol/L) or IKK-2 inhibitor BOT64 (10 μmol/L) for 24 hours, demonstrating that NFκB inhibition but not calcineurin inhibition abrogates PMA-induced *SIG15* transcription (****, *P* < 0.0001). **D,** Western blot analysis shows SIG15 expression in REH cells following 24 hours of stimulation with 81 nmol/L PMA alone or in combination with 10 μmol/L of the IKK-2 inhibitor BOT64. NFκB inhibition attenuates PMA-induced upregulation of SIG15. SIG15 was probed using the 1F7 mAb (NextCure). **E,***SIG15* expression from the B-ALL TARGET database correlated with protein kinase C β (*PRKCB*; *r* = 0.35; *P* < 0.0001, Pearson correlation), calcineurin catalytic subunit alpha (*PPP3CA*; *r* = 0.42; *P* < 0.0001), and IL1β (*IL1B*; *r* = 0.33; *P* < 0.0001, Pearson correlation). **F,** Western blot analysis shows increased SIG15 protein in REH cells stimulated for 24 hours with increasing doses of recombinant human CD40L. Densitometric analysis of SIG15 is quantified above. SIG15 was probed using the 1F7 mAb (NextCure).

Immunosuppressive cytokines, such as MCSF and IL10, have previously been demonstrated to induce Sig15 expression in macrophages (11, 16). Although we tested these and several other candidate cytokines for their effects on Sig15 expression after 24 hours of treatment in an immortalized B-ALL cell line, REH, only chemical stimulation with PMA strongly induced Sig15 expression (Fig. 2A; Supplementary Fig. S2A). This PMA-mediated induction was dependent upon Protein kinase C (PKC) activation, as *SIGLEC15* upregulation was abrogated when cells were stimulated for 24 hours with a combination of PMA and a pan-PKC inhibitor, Gö6983 (Fig. 2B). As PKC activation is upstream of both calcineurin/NFAT and NFκB activation, we used an IKK-2 inhibitor (BOT64) and a calcineurin inhibitor (Cyclosporin A; CSA) in combination with 24-hour PMA stimulation to demonstrate that PMA-induced upregulation of Sig15 expression is dependent upon NFκB activation (Fig. 2C and D). Analyses of gene expression from the ALL project of the TARGET Program demonstrated strong positive correlations of *SIG15* expression with markers of PKC, calcineurin, and NFκB activity in pediatric B-ALL samples (Fig. 2E). As CD40L is a major immunoregulatory molecule upstream of NFκB activation in mature B cells, we tested its role in stimulating Sig15 expression in B-ALL cells. We found that stimulation for 24 hours with CD40L increased levels of SIG15 (Fig. 2F), suggesting that this inducer of the B-cell NFκB signaling axis are involved in regulation of SIG15 in B-ALL. Because of its critical role in lymphocyte development and survival, constitutive or overactivation of the NFκB pathway is common in subsets of B-cell malignancies (28, 29), which may contribute to the pathological overexpression of Sig15.

**FIGURE 3:**
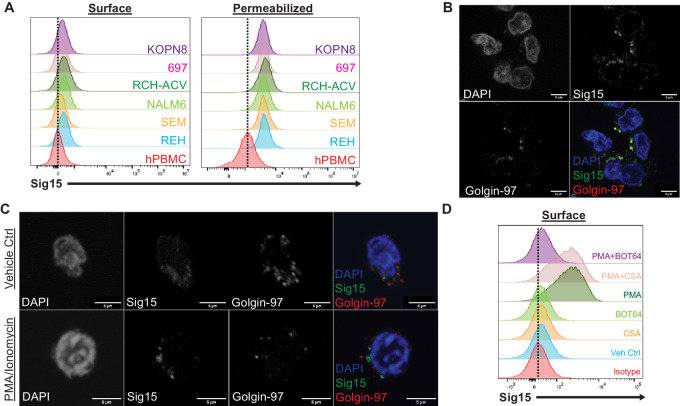
B-ALL–expressed Sig15 has dynamic subcellular localization. **A,** Flow cytometry of human B-ALL cell lines probed for SIG15 in both permeabilized and unpermeabilized (Surface) cells. Dotted line marks mean fluorescence intensity (MFI) of healthy PBMC signal (data representative of three independent experiments). Leukemia and PBMCs had modest surface expression of SIG15, while leukemia cells expressed higher intracellular SIG15 compared with PBMCs. SIG15 was probed using the NP159 mAb (NextCure). **B** and **C,** Immunofluorescence of REH cells staining for DAPI, Sig15, and Golgin-97. **B,** Imaging depicts limited surface localization of Sig15 and strong colocalization with the Golgi apparatus and TGN in unstimulated REH cells. **C,** Representative images depict REH cells treated for 24 hours with DMSO (vehicle control) or 81 nmol/L PMA and 1.3 μmol/L ionomycin. Stimulated REH cells show decreased colocalization of Sig15 signal with Golgin-97. SIG15 was probed using the Invitrogen polyclonal antibody. **D,** Flow cytometry of unpermeabilized REH cells treated with PMA with or without BOT64 for 6 hours, demonstrating that NFkB activation stimulates Sig15 localization at the cell surface (data representative of three independent experiments; dotted line represents MFI of isotype staining). SIG15 was probed using the NP159 mAb (NextCure) conjugated to Alexa Fluor 647.

Though we have demonstrated the high pathologic expression of Sig15 in B-ALL, none of the B-ALL cell lines expressed the primary Sig15 binding partner DAP12 (Fig. 1C) or alternative binding partner FcRγ (Supplementary Fig. S1C), which are required for downstream activation of Syk kinase and PI3K in myeloid cells (13). Binding of Siglec family members to these adapter proteins has been demonstrated to anchor these molecules at the cell membrane when they would otherwise turnover in the endosomal complex (30). Indeed, flow cytometry demonstrated that Sig15 appeared to be primarily localized intracellularly in human B-ALL cell lines (Fig. 3A). Furthermore, immunofluorescence revealed that intracellular Sig15 strongly colocalized with the Golgi apparatus and trans-Golgi network (TGN) marker, Golgin-97 (Fig. 3B), suggesting retention of Sig15 in the Golgi apparatus in B-ALL cells with limited trafficking to the membrane along the TGN. In accordance with other transmembrane proteins that possess regulated trafficking to the membrane, the intracellular domain of Sig15 contains a rapid internalization motif which can promote recycling of the protein back through the endosomal compartments (10), resulting in eventual localization in the lysosome (Supplementary Fig. S3A) unless otherwise stabilized at the membrane. Interestingly, PMA-induced NFκB activation for 24 hours reduced localization of Sig15 in the Golgi compartment (Supplementary Fig. S3B) and increased Sig15 at the surface of B-ALL cells (Fig. 3C and D). This increase in surface localization of Sig15 was mediated through NFκB activation, with peak surface expression at approximately 8 hours poststimulation (Supplementary Fig. S3C).

### Sig15 Circulates in the Plasma of Patients with B-ALL

**FIGURE 4:**
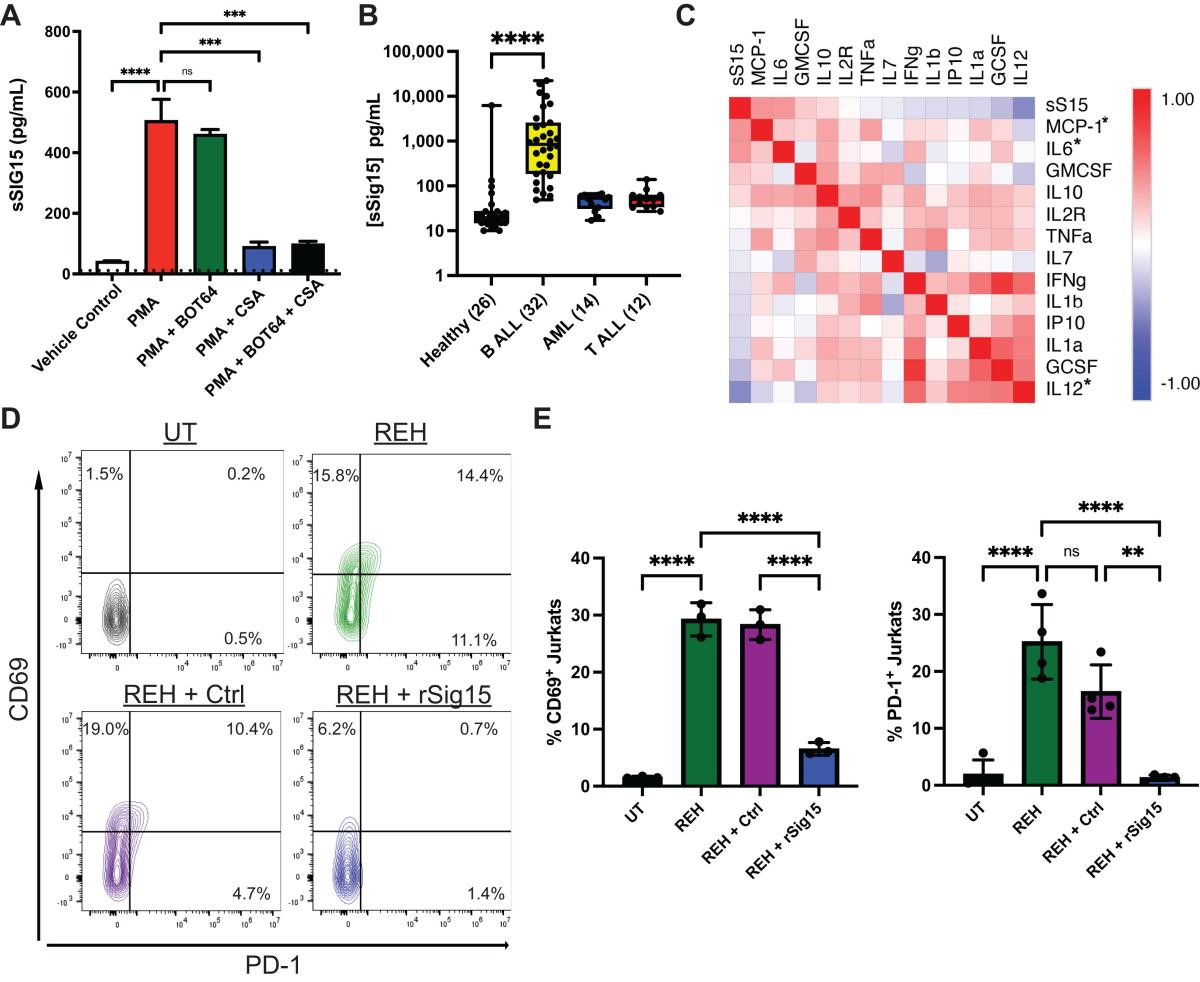
B-ALL cells release secreted/soluble Sig15 that circulates in plasma and inhibits activation of CAR-expressing Jurkat cells. **A,** sSIG15 detected via electrochemiluminescence (MSD) from the supernatant of REH cells treated for 24 hours with combinations of PMA (81 nmol/L), BOT64 (10 μmol/L), and CSA (1 μmol/L). Dotted line represents the assay limit of detection (10 pg/mL). PMA induces a calcineurin-dependent increase in the release of sSig15. **B,** sSIG15 detected via MSD from the plasma of pediatric patients with B-ALL, AML or T-ALL and healthy controls (sample size in parentheses). Pediatric patients with B-ALL showed higher plasma concentration of sSIG15 than healthy donors (****, *P* < 0.0001). **C,** Cytokines were measured in the plasma from children with leukemia using Luminex and compared with sSig15. Pearson correlation of a subset is depicted graphically using the nearest neighbor algorithm (*, *P* < 0.05). **D** and **E,** Jurkat cells expressing CD19-CAR constructs cultured alone (UT = untreated) or cocultured with CD19^+^ REH B-ALL cells (REH) at an effector:target ratio of 2:1 with or without 50 μg/mL protein control (Ctrl = human IgG) or recombinant Sig15 (rSig15) for 4 hours and stained for early activation markers CD69 and PD-1. **D,** Representative contour plots of flow cytometry data. **E,** Quantification of four independent experiments. Recombinant Sig15 inhibits early Jurkat activation when in coculture with B-ALL cells.

As the TGN along which Sig15 traffics is known to regulate vesicular secretion of proteins (31), and as other Siglec family member proteins have been demonstrated to exist in soluble form (32, 33), we hypothesized that B-ALL cells have the capacity to release a secreted or soluble form of Sig15 (sSig15). We detected sSig15 in the supernatant of REH cells in culture using MSD, which was increased with PMA stimulation (Fig. 4A). Combination treatment with the calcineurin inhibitor, cyclosporine A, demonstrated that calcineurin activation, downstream of PKC, regulates sSig15 release into the supernatant but not its mRNA expression level. We then assayed cryopreserved plasma from healthy subjects and pediatric patients with leukemia collected at the time of diagnosis for sSig15 levels, finding significantly higher sSig15 in the plasma of pediatric patients with B-ALL relative to healthy donors (Fig. 4B).

Though some members of the Siglec family proteins are known to circulate in plasma (32, 33), to our knowledge, this is the first demonstration of a circulating, secreted/soluble form of Sig15. Further analyses of circulating cytokine levels in these leukemia patient plasma samples via Luminex multiplex assay found a correlation between sSig15 and the cytokines MCP-1/CCL2 and IL6 (Fig. 4C), which have been demonstrated to support the formation of the proleukemic bone marrow microenvironment (34, 35). Notably, sSig15 strongly negatively correlated with IL12 levels, a proinflammatory cytokine with potent antileukemia activity (7, 24). To address the functional capacity of sSig15 to suppress immune activity, we treated CD19-CAR expressing Jurkat cells with recombinant human Sig15 in coculture with a CD19^+^ B-ALL cell line. Recombinant Sig15 significantly suppressed the early activation of the CAR^+^ Jurkat cells (Fig. 4D and E), suggesting that sSig15 may directly suppress T-cell activation and function.

### Sig15 is Required for Immune Evasion by B-ALL Cells

**FIGURE 5:**
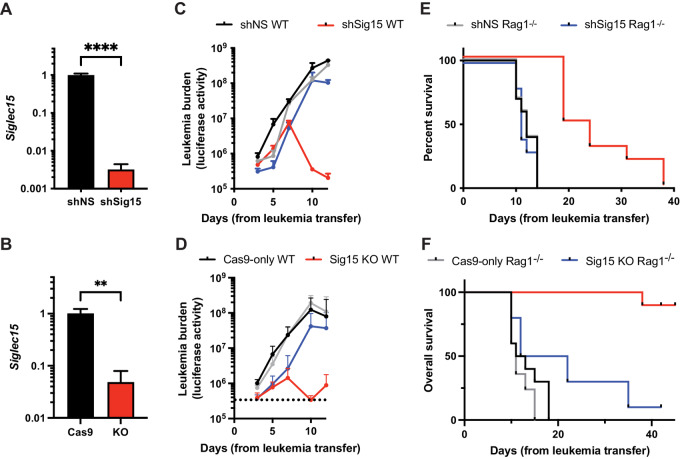
Sig15 is required for adaptive immune escape in murine B-ALL. qRT-PCR shows genetic knockdown of *Siglec15* expression in a murine model of B-ALL by shRNA (shSig15; **A**) or knocked out using CRISPR/Cas9 (Sig15 KO; **B**). Data are normalized to the expression levels of a non-silencing vector control (shNS) or Cas9-expressing cells without gRNA (Cas9). **C** and **D,** Unirradiated WT or *Rag1^−^^/^^−^* C57BL6 mice were injected via tail vein with 5 × 10^5^ control or Sig15-deficient leukemia cells. Luciferase signal over time represents disease burden as measured via IVIS imaging over 14 days. Kaplan–Meier curve shows prolonged survival of WT recipients of Sig15-deficient leukemia. **E,***P* < 0.0001; shNS WT versus shSig15 WT, Mantel–Cox log-rank test; *n* = 10 per group from two independent experiments. **F,***P* < 0.0001, Cas9-only WT versus Sig15 KO WT; *n* = 10 per group from two independent experiments.

With data supporting the pathologic expression and immunosuppressive effects of Sig15 in B-ALL, we next sought to characterize the effects of Sig15 ablation in a murine model of B-ALL. We performed shRNA knockdown and CRISPR-mediated deletion of Sig15 in a well-characterized *BCR-ABL1^+^ Arf^−^^/^^−^* murine B-ALL cell line (Fig. 5A and B; ref. 19). In immune-competent WT and immune-deficient *Rag1^−^^/^^−^* recipients, the control leukemias progressed rapidly, necessitating euthanasia of all recipients within 2–3 weeks of leukemia transfer (Fig 5C and D; Supplementary Fig. S4A). In stark contrast, after 7–10 days, the leukemia burden in WT recipients of Sig15-deficient leukemia dramatically declined to below the limit of detection, and the WT recipients of Sig15-deficient leukemia survived significantly longer than recipients of the control leukemias (Fig. 5E and F). Sig15 ablation did not alter basal apoptosis levels nor proliferation rates of these cells (Supplementary Fig. S4B and S4C). The initial increase in leukemia burden in immunocompetent recipients of Sig15-deficient leukemia, followed by regression around day 7, is consistent with well-described kinetics of T-cell responses *in vivo* (36).

**FIGURE 6:**
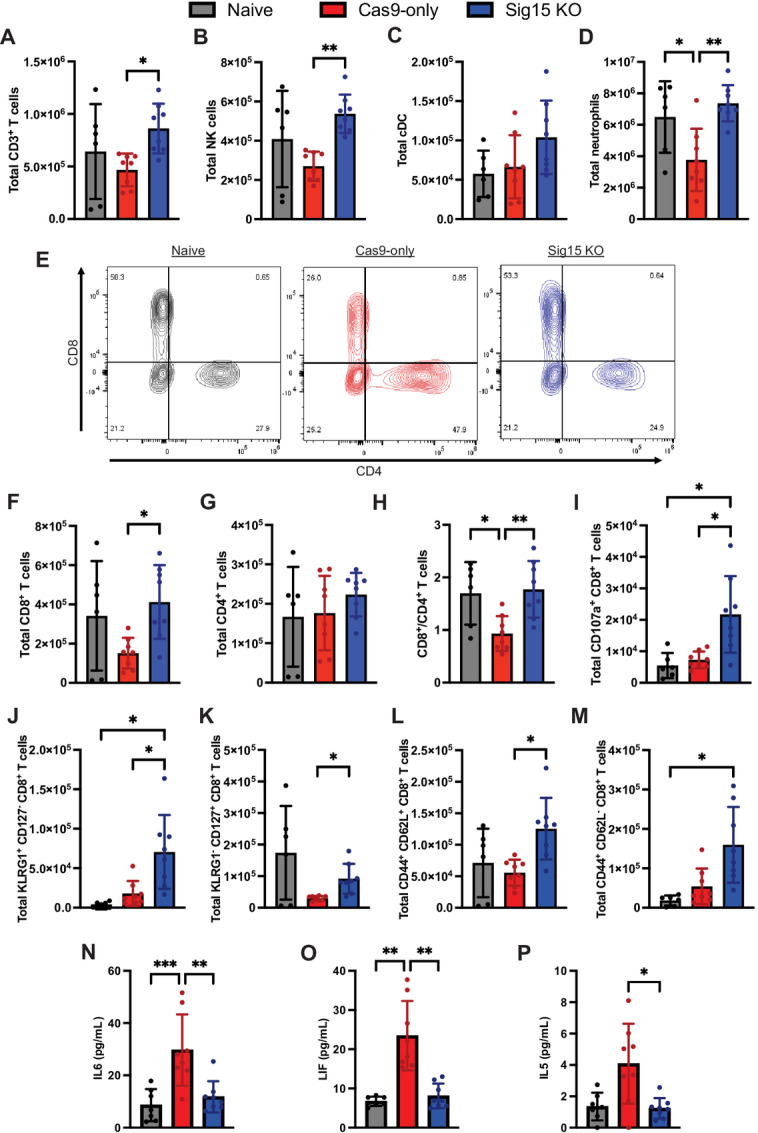
Sig15 promotes an immunosuppressive tumor microenvironment in B-ALL. **A–M,** Unirradiated WT C57BL6 mice were injected via tail vein with 5 × 10^5^ Cas9-only (*n* = 8 from two independent experiments) or Sig15 KO (*n* = 8) leukemia. Bone marrow was harvested 7 days later for highly dimensional flow cytometry of non-leukemia bone marrow populations. Healthy, leukemia-naïve mice (Naive) were included as controls (*n* = 6). CD3^+^ T cells (**A**), NK cells (**B**), classical dendritic cells (cDC; **C**), and neutrophils (**D**) were variably present at higher totals in the bone marrow of Sig15 KO recipient mice compared with control leukemia. **E,** Representative contour plots of T-cell populations. Proportions of CD8^+^ (**F**) but not CD4^+^ (**G**) T cells were higher in Sig15 KO recipients than control leukemia, increasing the CD8^+^/CD4^+^ T-cell ratio (**H**) to levels comparable with Naive mice. **I,** Degranulated CD8^+^ T cells were higher in Sig15 KO recipient mice, as were SLEC (**J**) and MPEC (**K**) CD8^+^ T-cell populations. Early memory-like populations consistent with central (**L**) and effector memory (**M**) CD8^+^ T-cell populations were also higher in Sig15 KO recipients. **N–P,** From the same experiments, bone marrow supernatant from control and Sig15 KO leukemia recipient mice was analyzed via 44-plex cytokine/chemokine assay. Levels of IL6 (**N**), LIF (**O**), and IL5 (**P**) were all significantly lower in the bone marrow of Sig15 KO recipients as compared with control leukemia (*, *P* < 0.05; **, *P* < 0.01; ***, *P* < 0.001; ****, *P* < 0.0001). Sig15 expression on B-ALL cells suppresses CD8^+^ T-cell expansion and activation and contributes overall to formation of a more proleukemia bone marrow microenvironment.

To better understand the effects of Sig15 on immune response to B-ALL, we engrafted immunocompetent recipients with control or Sig15 knockout (Sig15 KO) leukemia and harvested bone marrow at day 7 postengraftment for immunophenotyping by flow cytometry (Supplementary Fig. S5). The numbers of bone marrow CD3^+^ T cells, natural killer (NK) cells, and neutrophils were all significantly higher in Sig15 KO leukemia recipients compared with control leukemia (Fig. 6A and D), characterizing a more robust antileukemia immune response. Among T-cell subsets (Fig. 6E), CD8^+^ cytotoxic T cells (CTL) were significantly expanded in Sig15 KO recipients, making the proportion of CD8:CD4 T cells comparable with levels in mice without leukemia (Fig. 6F–H). In addition, markers of T-cell activation and degranulation were significantly increased in CD8^+^ T cells in Sig15 KO recipients (Fig. 6I; Supplementary Fig. S6A and S6B), while activation markers were more variably elevated in NK cells, classical dendritic cells, and neutrophils (Supplementary Fig. S6E–S6J). Those CD8^+^ T-cell populations expected to be critical in the early stages of immunologic control, such as short-lived effector cell (SLEC) and memory precursor effector CD8^+^ cells (MPEC) populations, were significantly higher (Fig. 6J and K) along with precursors to central and effector memory T cells (Fig. 6L and M), suggesting both heightened early response and long-term immunologic benefit. Finally, we observed a significant decrease in IL6, LIF, and IL5 in the bone marrow of Sig15 KO recipients (Fig. 6N–P). LIF and IL5 have been demonstrated to mediate antigen-specific immune tolerance through induction and modulation of regulatory T cells (37, 38), while IL6 is known to suppress CD8^+^ T cell–mediated clearance of B-ALL in response to chemotherapy (39). Thus, Sig15 expressed by B-ALL cells may contribute to the formation of a more immunologically favorable leukemia bone marrow niche via these factors.

## Discussion

The results of our investigation support Sig15 as a critical immunomodulator in suppression of T cell–mediated response to leukemia and disease clearance in B-ALL. Sig15 is widely expressed across hematologic malignancies at consistently higher levels compared with healthy controls in primary samples and in B-ALL, T-ALL, and DLBCL cell lines. This overexpression of Sig15 compared with paired healthy tissue has been demonstrated in several solid tumors, including NSCLC (11) and clear-cell renal cell carcinoma (17), but has yet to be described in ALL. Our findings in primary pediatric populations also corroborate the published overexpression of Sig15 in other diseases such as osteosarcoma (18) and AML (25). The scope of malignancies which overexpress this molecule provide support for a selective advantage to pathologic upregulation of Sig15 by cancer cells and tumor-associated immune cells.

To date, most publications on Sig15 have characterized its role in the context of mature osteoclasts, which have shared precursors with myeloid cells (40), as well as tumor-infiltrating myeloid cells and myeloid blood cancers. These myeloid-derived cells often express abundant amounts of the adaptor proteins DAP12 and FcRy (41), which can couple with sialic acid–bound Sig15 to recruit Syk kinase and PI3K to their ITAM domains (13, 16), allowing for intracellular signal transduction. As we did not detect these adaptor molecules in lymphoid blood cancers, this intracellular signaling axis is likely not active, which could suggest that ALL-expressed Sig15 does not function primarily as a receptor in ALL. Rather, alongside data from others demonstrating inhibition of T-cell activity with recombinant Sig15 (11), our observations of dynamic Sig15 surface localization and circulating sSig15 could suggest the possibility of a primary function as a ligand, locally and systemically. Indeed, recombinant Sig15 attenuated activation of CD19-CAR–expressing Jurkat cells, when cultured with CD19^+^ B-ALL cells. Though recent studies have demonstrated binding of recombinant Sig15 protein to partners such as CD44 (42), the canonical receptor for cancer-expressed Sig15, particularly as expressed on T-cell populations, remains to be defined. An important consideration of all of the data regarding extracellular Sig15 is that the studied recombinant forms are tagged with His or Fc sequences that may alter function. Characterization of the sequence and structure of sSig15 will be critical to fully understand its physiologic function.

Lack of expression of adaptor molecules DAP12 and FcRy in lymphoblastic leukemias could also contribute to the select trafficking and localization of Sig15 at the membrane in ALL. We observed that Sig15 localized primarily intracellularly in B-ALL cell lines unless stimulated with an inducer of NFκB, which increased both total Sig15 and cell surface expression. As a cell surface molecule, Sig15 has been demonstrated to be localized on the surface of mature osteoclasts (13, 43) as well as subsets of macrophages (11). Some subsets of DC-SIGN–expressing macrophages and dendritic cells, however, have been shown to have intracellular Sig15 (10), which suggests dynamic regulation of the expression and localization of Sig15 in these cells and in B-ALL, the latter being mediated through NFκB activation.

NFκB has been demonstrated in some contexts to regulate membrane protein trafficking through activation of downstream mediator AKT (44) or through direct regulation of expression of intracellular trafficking proteins Rab10 and Acp5 (45). Though the mechanism by which NFκB activation regulates Sig15 localization in ALL is still being explored, our analysis revealed Sig15 on the surface of many primary B-ALL leukemia samples from the bone marrow which was not seen in comparable healthy donor B cell progenitors. As the NFκB pathway is often active in B-ALL cells and enhanced in the leukemia bone marrow niche (28, 29, 46), this could drive both the pathologic expression and surface localization of Sig15 in B-ALL. Our findings also suggest a role for the CD40L-CD40 signaling axis, upstream of NFκB, in regulating Sig15 expression. CD40L is upregulated during T-cell activation, and its binding with CD40 has been demonstrated to result in upregulation of PD-L1 in tumor-infiltrating macrophages (47). It is thus possible that CD40L in the B-ALL bone marrow niche may locally enhance Sig15 expression through NFκB activation, which could further suggest a role for Sig15 in regulating inflammation and T cell activation alongside other immune checkpoint molecules.

Immunofluorescent imaging revealed that intracellular Sig15 strongly colocalized with Golgin-97 signal in human B-ALL cells. The protein sequence of Sig15 also contains a signal peptide motif often present in secreted proteins (48), which together suggests these cells have the capacity to release Sig15 through the secretory pathway. Other Siglec family member proteins are also known to be released as soluble proteins through expression of alternative isoforms lacking transmembrane domains or undergoing ectodomain shedding at the membrane (33, 49). Though the method by which sSig15 is released from B-ALL cells is still in ingoing investigation, sSig15 was in fact detectable both in the supernatant of human B-ALL cell lines and in the plasma of pediatric patients with B-ALL. This is to our knowledge the first demonstration of a secreted/soluble form of Sig15 in humans. In contrast to the regulatory axis of Sig15 expression, the release of sSig15 appears to be regulated by calcineurin activation. Calcineurin has been demonstrated through activation of NFAT and other downstream targets to regulate vesicular trafficking and secretion of proteins (50) as well as the expression and activity of extracellular proteases such as cathepsins responsible for protein shedding (51). sSig15 in pediatric patients with B-ALL was found to negatively correlate with antileukemia cytokines such as IL12 and positively correlate with immunosuppressive factors such as MCP-1/CCL2 and IL6, suggesting sSig15 is one component of a complex immunosuppressive local and systemic microenvironment in B-ALL. Our demonstrations of the capacity of recombinant Sig15 to suppress early activation in a CAR-T cell model further suggest its functionality in directly regulating immunosuppression. Nonetheless, whether cell surface or extracellular Sig15 is most functional in B-ALL remains to be demonstrated.

Although higher Sig15 mRNA levels are associated with prolonged event-free survival in the cohort that we analyzed, these data need to be interpreted cautiously, as the cohort is enriched in those with early relapse and is clinically and molecularly diverse. Moreover, the protein levels of Sig15, cell surface or circulating, are not known. Most importantly, Sig15 was found to be critical for the capacity of B-ALL cells to evade immune clearance in a murine model of B-ALL. Ablation of Sig15 expression in these leukemia cells resulted in increased expansion and activation of multiple immune populations in the bone marrow, including NK cells, classical dendritic cells, neutrophils, and CD8^+^ T cells. For the former, the CD8^+^/CD4^+^ T-cell ratio was returned to basal levels observed in mice without leukemia, and early effector populations were highly expanded at the day 7 timepoint in the bone marrow of Sig15 KO leukemia recipients. These SLEC and MPEC populations are predicted to be the primary effectors at the peak of acute inflammatory response (52), suggesting that their expansion in Sig15 KO leukemia recipients facilitates greater leukemia control. There were also notable reductions in the leukemia-induced immunosuppressive cytokine milieu of Sig15 KO leukemia recipients, marked by decreases in IL6, LIF, and IL5 that all contribute to a more immune-privileged bone marrow niche. These results, combined with our data in primary human samples, suggest Siglec-15 is a novel, potent immunosuppressive molecule active in leukemia that may be targeted therapeutically to activate CTLs against leukemia.

## Supplementary Material

Supplemental Figure 1 Pathological overexpression of Sig15 in leukemia.

Supplemental Figure 2 Sig15 is regulated by NF-κB activation in B-ALL.

Supplemental Figure 3 Sig15 localizes to the Golgi apparatus and TGN in B-ALL.

Supplemental Figure 4 Sig15 is required for immune evasion by murine B-ALL.

Supplemental Figure 5 Flow cytometry gating schemes.

Supplemental Figure 6 Sig15 ablation in murine B-ALL promotes an adaptive immune response against leukemia.

Supplemental Table 1 All gRNA, shRNA, and primer sequences for genetic modification and RT-qPCR analysis.

Supplemental Table 2 All antibodies used for flow cytometry analysis.

## References

[1] Howlader N , Noone AM, Krapcho M, Miller D, Brest A, Yu M, . SEER cancer statistics review, 1975–2018, National Cancer Institute, Bethesda, MD.

[2] PDQ Pediatric Treatment Editorial Board. Childhood acute lymphoblastic leukemia treatment (PDQ®): health professional version. PDQ Cancer Information Summaries; 2021.

[3] Li Y , Gupta G, Molofsky A, Xie Y, Shihabi N, McCormick J, . B lymphoblastic leukemia/lymphoma with burkitt-like morphology and IGH/MYC rearrangement: report of 3 cases in adult patients. Am J Surg Pathol 2018;42:269–76. 29112016 10.1097/PAS.0000000000000982 PMC5762415

[4] Hunger SP , Raetz E. How I treat relapsed acute lymphoblastic leukemia in the pediatric population. Blood 2020;136:1803–12. 32589723 10.1182/blood.2019004043

[5] Grupp SA , Maude SL, Shaw PA, Aplenc R, Barrett DM, Callahan C, . Durable remissions in children with relapsed/refractory ALL treated with T cells engineered with a CD19-targeted chimeric antigen receptor (CTL019). Blood 2015;126:681.

[6] Gore L , Locatelli F, Zugmaier G, Handgretinger R, O'Brien MM, Bader P, . Survival after blinatumomab treatment in pediatric patients with relapsed/refractory B-cell precursor acute lymphoblastic leukemia. Blood Cancer J 2018;8:80. 30190453 10.1038/s41408-018-0117-0 PMC6127096

[7] Rabe JL , Gardner L, Hunter R, Fonseca JA, Dougan J, Gearheart CM, . IL-12 abrogates calcineurin-dependent immune evasion during leukemia progression. Cancer Res 2019;79:3702–13. 31142509 10.1158/0008-5472.CAN-18-3800 PMC6889000

[8] Crocker PR , Paulson JC, Varki A. Siglecs and their roles in the immune system. Nat Rev Immunol 2007;7:255–66. 17380156 10.1038/nri2056

[9] Bornhöfft KF , Goldammer T, Rebl A, Galuska SP. Siglecs: a journey through the evolution of sialic acid-binding immunoglobulin-type lectins. Dev Comp Immunol 2018;86:219–31. 29751010 10.1016/j.dci.2018.05.008

[10] Angata T , Tabuchi Y, Nakamura K, Nakamura M. Siglec-15: an immune system Siglec conserved throughout vertebrate evolution. Glycobiology 2007;17:838–46. 17483134 10.1093/glycob/cwm049

[11] Wang J , Sun J, Liu LN, Flies DB, Nie X, Toki M, . Siglec-15 as an immune suppressor and potential target for normalization cancer immunotherapy. Nat Med 2019;25:656–66. 30833750 10.1038/s41591-019-0374-x PMC7175920

[12] Hiruma Y , Hirai T, Tsuda E. Siglec-15, a member of the sialic acid-binding lectin, is a novel regulator for osteoclast differentiation. Biochem Biophys Res Commun 2011;409:424–9. 21586272 10.1016/j.bbrc.2011.05.015

[13] Kameda Y , Takahata M, Komatsu M, Mikuni S, Hatakeyama S, Shimizu T, . Siglec-15 regulates osteoclast differentiation by modulating RANKL-induced phosphatidylinositol 3-kinase/Akt and Erk pathways in association with signaling adaptor DAP12. J Bone Miner Res 2013;28:2463–75. 23677868 10.1002/jbmr.1989

[14] Ishida-Kitagawa N , Tanaka K, Bao X, Kimura T, Miura T, Kitaoka Y, . Siglec-15 protein regulates formation of functional osteoclasts in concert with DNAX-activating protein of 12 kDa (DAP12). J Biol Chem 2012;287:17493–502. 22451653 10.1074/jbc.M111.324194 PMC3366812

[15] Hiruma Y , Tsuda E, Maeda N, Okada A, Kabasawa N, Miyamoto M, . Impaired osteoclast differentiation and function and mild osteopetrosis development in Siglec-15-deficient mice. Bone 2013;53:87–93. 23238125 10.1016/j.bone.2012.11.036

[16] Takamiya R , Ohtsubo K, Takamatsu S, Taniguchi N, Angata T. The interaction between Siglec-15 and tumor-associated sialyl-Tn antigen enhances TGF-beta secretion from monocytes/macrophages through the DAP12-Syk pathway. Glycobiology 2013;23:178–87. 23035012 10.1093/glycob/cws139

[17] Liu Y , Li X, Zhang C, Zhang H, Huang Y. LINC00973 is involved in cancer immune suppression through positive regulation of Siglec-15 in clear-cell renal cell carcinoma. Cancer Sci 2020;111:3693–704. 32780490 10.1111/cas.14611 PMC7541001

[18] Fan M-K , Zhang G-C, Chen W, Qi L-L, Xie M-F, Zhang Y-Y, . Siglec-15 promotes tumor progression in osteosarcoma via DUSP1/MAPK pathway. Front Oncol 2021;11:710689. 34336699 10.3389/fonc.2021.710689 PMC8322944

[19] Williams RT , Roussel MF, Sherr CJ. Arf gene loss enhances oncogenicity and limits imatinib response in mouse models of Bcr-Abl-induced acute lymphoblastic leukemia. Proc Natl Acad Sci U S A 2006;103:6688–93. 16618932 10.1073/pnas.0602030103 PMC1440588

[20] Williams RT , den Besten W, Sherr CJ. Cytokine-dependent imatinib resistance in mouse BCR-ABL+, Arf-null lymphoblastic leukemia. Genes Dev 2007;21:2283–7. 17761812 10.1101/gad.1588607 PMC1973142

[21] Boulos N , Mulder HL, Calabrese CR, Morrison JB, Rehg JE, Relling MV, . Chemotherapeutic agents circumvent emergence of dasatinib-resistant BCR-ABL kinase mutations in a precise mouse model of Philadelphia chromosome-positive acute lymphoblastic leukemia. Blood 2011;117:3585–95. 21263154 10.1182/blood-2010-08-301267 PMC3072880

[22] Rafiq S , Yeku OO, Jackson HJ, Purdon TJ, van Leeuwen DG, Drakes DJ, . Targeted delivery of a PD-1-blocking scFv by CAR-T cells enhances anti-tumor efficacy *in vivo*. Nat Biotechnol 2018;36:847–56. 30102295 10.1038/nbt.4195 PMC6126939

[23] Schindelin J , Arganda-Carreras I, Frise E, Kaynig V, Longair M, Pietzsch T, . Fiji: an open-source platform for biological-image analysis. Nat Methods 2012;9:676–82. 22743772 10.1038/nmeth.2019 PMC3855844

[24] Hunter R , Imbach KJ, Zhou C, Dougan J, Hamilton JAG, Chen KZ, . B-cell acute lymphoblastic leukemia promotes an immune suppressive microenvironment that can be overcome by IL-12. Sci Rep 2022;12:11870. 35831470 10.1038/s41598-022-16152-z PMC9279427

[25] Cao H , Neerincx A, de Bono B, Lakner U, Huntington C, Elvin J, . Sialic acid-binding immunoglobulin-like lectin (Sigelac)-15 is a rapidly internalised cell-surface antigen expressed by acute myeloid leukaemia cells. Br J Haematol 2021;193:946–50. 33951750 10.1111/bjh.17496

[26] Elvin J , Huntington C, Trowsdale J, Barrow A, Cao H. Anti-Siglec15 antibodies and uses thereof. Cambridge Enterprise Limited, MedImmune Limited, assignee, 2015/0037356 A1; 2015.

[27] Sun C , Chang L, Zhu X. Pathogenesis of ETV6/RUNX1-positive childhood acute lymphoblastic leukemia and mechanisms underlying its relapse. Oncotarget 2017;8:35445–59. 28418909 10.18632/oncotarget.16367 PMC5471068

[28] Montaño A , Ordoñez JL, Alonso-Pérez V, Hernández-Sánchez J, Santos S, González T, . ETV6/RUNX1 fusion gene abrogation decreases the oncogenicity of tumour cells in a preclinical model of acute lymphoblastic leukaemia. Cells 2020;9:215. 31952221 10.3390/cells9010215 PMC7017301

[29] Hsieh M-Y , Van Etten RA. IKK-dependent activation of NF-κB contributes to myeloid and lymphoid leukemogenesis by BCR-ABL1. Blood 2014;123:2401–11. 24464015 10.1182/blood-2014-01-547943 PMC3983614

[30] Chang Y-C , Nizet V. The interplay between Siglecs and sialylated pathogens. Glycobiology 2014;24:818–25. 24996821 10.1093/glycob/cwu067 PMC4168292

[31] Derby MC , van Vliet C, Brown D, Luke MR, Lu L, Hong W, . Mammalian GRIP domain proteins differ in their membrane binding properties and are recruited to distinct domains of the TGN. J Cell Sci 2004;117:5865–74. 15522892 10.1242/jcs.01497

[32] Legrand F , Cao Y, Wechsler JB, Zhu X, Zimmermann N, Rampertaap S, . Sialic acid-binding immunoglobulin-like lectin (Siglec) 8 in patients with eosinophilic disorders: receptor expression and targeting using chimeric antibodies. J Allergy Clin Immunol. 2019;143:2227–2237.e10. 30543818 10.1016/j.jaci.2018.10.066 PMC6556424

[33] Huang PCJ , Low P-Y, Wang I, Hsu S-TD, Angata T. Soluble Siglec-14 glycan-recognition protein is generated by alternative splicing and suppresses myeloid inflammatory responses. J Biol Chem 2018;293:19645–58. 30377253 10.1074/jbc.RA118.005676 PMC6314137

[34] Isidro-Hernández M , Mayado A, Casado-García A, Martínez-Cano J, Palmi C, Fazio G, . Inhibition of inflammatory signaling in Pax5 mutant cells mitigates B-cell leukemogenesis. Sci Rep 2020;10:19189. 33154497 10.1038/s41598-020-76206-y PMC7644722

[35] de Vasconcellos JF , Laranjeira AB, Zanchin NI, Otubo R, Vaz TH, Cardoso AA, . Increased CCL2 and IL-8 in the bone marrow microenvironment in acute lymphoblastic leukemia. Pediatr Blood Cancer 2011;56:568–77. 21298741 10.1002/pbc.22941

[36] Akondy RS , Fitch M, Edupuganti S, Yang S, Kissick HT, Li KW, . Origin and differentiation of human memory CD8 T cells after vaccination. Nature 2017;552:362–7. 29236685 10.1038/nature24633 PMC6037316

[37] Nasef A , Mazurier C, Bouchet S, François S, Chapel A, Thierry D, . Leukemia inhibitory factor: role in human mesenchymal stem cells mediated immunosuppression. Cell Immunol 2008;253:16–22. 18639869 10.1016/j.cellimm.2008.06.002

[38] Tran GT , Hodgkinson SJ, Carter NM, Verma ND, Plain KM, Boyd R, . IL-5 promotes induction of antigen-specific CD4+CD25+ T regulatory cells that suppress autoimmunity. Blood 2012;119:4441–50. 22310911 10.1182/blood-2011-12-396101

[39] Bent EH , Millán-Barea LR, Zhuang I, Goulet DR, Fröse J, Hemann MT. Microenvironmental IL-6 inhibits anti-cancer immune responses generated by cytotoxic chemotherapy. Nat Commun 2021;12:6218. 34711820 10.1038/s41467-021-26407-4 PMC8553783

[40] Xiao Y , Zijl S, Wang L, de Groot DC, van Tol MJ, Lankester AC, . Identification of the common origins of osteoclasts, macrophages, and dendritic cells in human hematopoiesis. Stem Cell Reports 2015;4:984–94. 26004632 10.1016/j.stemcr.2015.04.012 PMC4471835

[41] Lanier LL . DAP10- and DAP12-associated receptors in innate immunity. Immunol Rev 2009;227:150–60. 19120482 10.1111/j.1600-065X.2008.00720.x PMC2794881

[42] Chang L , Chen YJ, Fan CY, Tang CJ, Chen YH, Low PY, . Identification of siglec ligands using a proximity labeling method. J Proteome Res 2017;16:3929–41. 28899088 10.1021/acs.jproteome.7b00625

[43] Stuible M , Moraitis A, Fortin A, Saragosa S, Kalbakji A, Filion M, . Mechanism and function of monoclonal antibodies targeting siglec-15 for therapeutic inhibition of osteoclastic bone resorption. J Biol Chem 2014;289:6498–512. 24446437 10.1074/jbc.M113.494542 PMC3945315

[44] Sommermann TG , O'Neill K, Plas DR, Cahir-McFarland E. IKKβ and NF-κB transcription govern lymphoma cell survival through AKT-induced plasma membrane trafficking of GLUT1. Cancer Res 2011;71:7291–300. 21987722 10.1158/0008-5472.CAN-11-1715 PMC3228879

[45] Liu J , Xiang J, Li X, Blankson S, Zhao S, Cai J, . NF-κB activation is critical for bacterial lipoprotein tolerance-enhanced bactericidal activity in macrophages during microbial infection. Sci Rep 2017;7:40418. 28079153 10.1038/srep40418 PMC5227741

[46] Balandrán JC , Purizaca J, Enciso J, Dozal D, Sandoval A, Jiménez-Hernández E, . Pro-inflammatory-related loss of CXCL12 niche promotes acute lymphoblastic leukemic progression at the expense of normal lymphopoiesis. Front Immunol 2017;7:666. 28111575 10.3389/fimmu.2016.00666 PMC5216624

[47] Zippelius A , Schreiner J, Herzig P, Müller P. Induced PD-L1 expression mediates acquired resistance to agonistic anti-CD40 treatment. Cancer Immunol Res 2015;3:236–44. 25623164 10.1158/2326-6066.CIR-14-0226

[48] Blobel G , Dobberstein B. Transfer of proteins across membranes. I. Presence of proteolytically processed and unprocessed nascent immunoglobulin light chains on membrane-bound ribosomes of murine myeloma. J Cell Biol 1975;67:835–51. 811671 10.1083/jcb.67.3.835 PMC2111658

[49] Ito T , Ishigami M, Matsushita Y, Hirata M, Matsubara K, Ishikawa T, . Secreted ectodomain of SIGLEC-9 and MCP-1 synergistically improve acute liver failure in rats by altering macrophage polarity. Sci Rep 2017;7:44043. 28272428 10.1038/srep44043 PMC5358744

[50] Ye L , Gratton A, Hannan NJ, Cannon P, Deo M, Palmer KR, . Nuclear factor of activated T-cells (NFAT) regulates soluble fms-like tyrosine kinase-1 secretion (sFlt-1) from human placenta. Placenta 2016;48:110–8. 27871461 10.1016/j.placenta.2016.10.013

[51] Balkan W , Martinez AF, Fernandez I, Rodriguez MA, Pang M, Troen BR. Identification of NFAT binding sites that mediate stimulation of cathepsin K promoter activity by RANK ligand. Gene 2009;446:90–8. 19563866 10.1016/j.gene.2009.06.013

[52] Sarkar S , Kalia V, Haining WN, Konieczny BT, Subramaniam S, Ahmed R. Functional and genomic profiling of effector CD8 T cell subsets with distinct memory fates. J Exp Med 2008;205:625–40. 18316415 10.1084/jem.20071641 PMC2275385

[53] Haferlach T , Kohlmann A, Wieczorek L, Basso G, Kronnie GT, Bene MC, . Clinical utility of microarray-based gene expression profiling in the diagnosis and subclassification of leukemia: report from the International Microarray Innovations in Leukemia Study Group. J Clin Oncol 2010;28:2529–37. 20406941 10.1200/JCO.2009.23.4732 PMC5569671

