# Supplementary material for: Siglec-15 Promotes Evasion of Adaptive Immunity in B-cell Acute Lymphoblastic Leukemia
Source: Cancer Res Commun. 2023 Jul 17;3(7):1248–59. doi: 10.1158/2767-9764.CRC-23-0056 (PMC10351425; doi:10.1158/2767-9764.CRC-23-0056)
Supplement: Supplemental Figure 1 — Pathological overexpression of Sig15 in leukemia. [file crc-23-0056-s01.pdf]

# Supplementary Figure 1

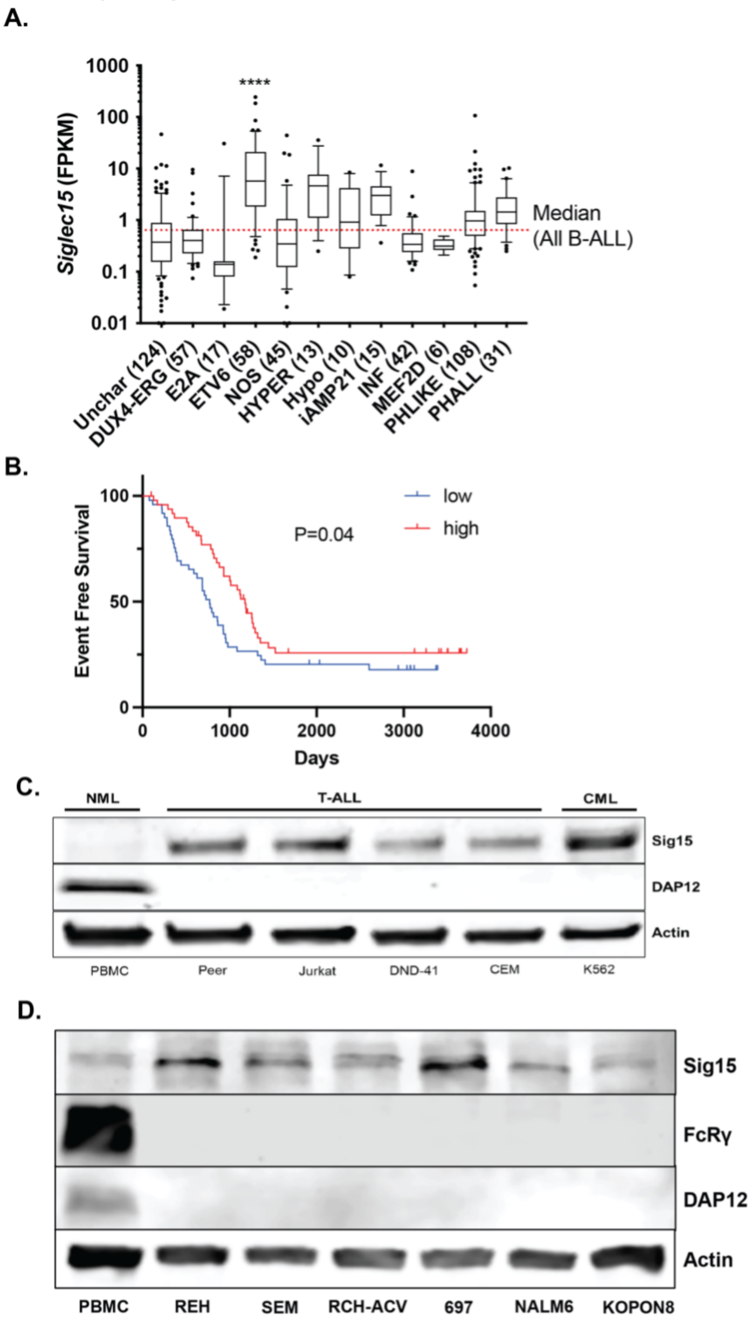


**Supplementary Figure 1. Pathological overexpression of Sig15 in leukemia. A.** Relative *SIGLEC15* expression from a panel of B-ALL genetic subtypes from the St. Jude PeCan database. Dotted red line indicates median expression across all subtypes. **B.** Kaplan-Meier curve of children with B-ALL with high v. low SIGLEC15 mRNA in the bone marrow at the time of diagnosis. **C.** Western blot analysis shows higher SIGLEC15 expression across a panel of T cell acute lymphoblastic leukemia (T-ALL) and a single chronic myelogenous leukemia (CML) cell line compared to normal peripheral blood mononuclear cells (NML). **D.** Western blot analysis shows a lack of expression of Sig15 binding partners DAP12 and FcRγ in a panel of human B-ALL cell lines compared to a healthy donor PBMC control.
