# Supplementary material for: Siglec-15 Promotes Evasion of Adaptive Immunity in B-cell Acute Lymphoblastic Leukemia
Source: Cancer Res Commun. 2023 Jul 17;3(7):1248–59. doi: 10.1158/2767-9764.CRC-23-0056 (PMC10351425; doi:10.1158/2767-9764.CRC-23-0056)
Supplement: Supplemental Figure 2 — Sig15 is regulated by NF-κB activation in B-ALL. [file crc-23-0056-s02.pdf]

Supplementary Figure 2

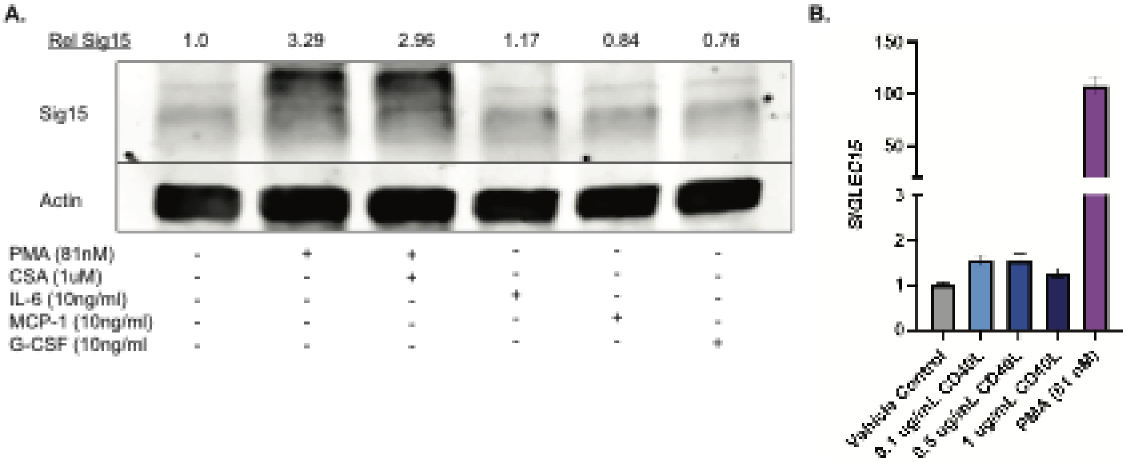

Supplementary Figure 2. Sig15 is regulated by NF-κB activation in B-ALL. A. Western blot analysis shows upregulated SIG15 expression in REH cells stimulated for 24 hours with 81 nM PMA which remains unaffected by addition of 1 uM Cyclosporin A. Recombinant IL-6, MCP-1, and G-CSF did not change SIG15 expression. Densitometric analysis of SIG15 is quantified above. **B.** qPCR of REH cells treated with increasing concentrations of recombinant human CD40L or PMA (81 nM) for 24 hours. CD40L does not induce an increase in *SIG15* expression at the RNA level in B-ALL cells.
