# Supplementary material for: Siglec-15 Promotes Evasion of Adaptive Immunity in B-cell Acute Lymphoblastic Leukemia
Source: Cancer Res Commun. 2023 Jul 17;3(7):1248–59. doi: 10.1158/2767-9764.CRC-23-0056 (PMC10351425; doi:10.1158/2767-9764.CRC-23-0056)
Supplement: Supplemental Figure 3 — Sig15 localizes to the Golgi apparatus and TGN in B-ALL. [file crc-23-0056-s03.pdf]

Supplementary Figure 3

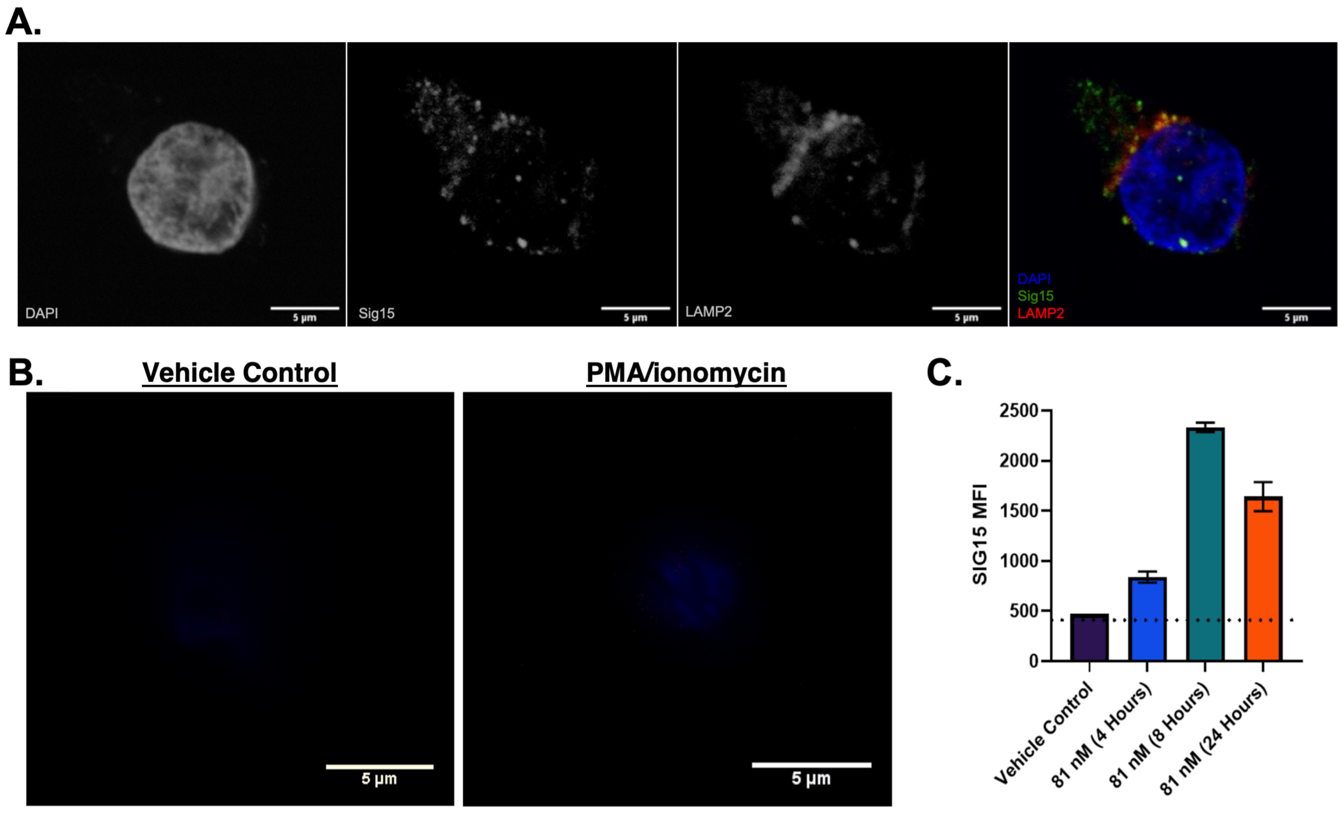

Supplementary Figure 3. Sig15 localizes to the Golgi apparatus and TGN in B-ALL.

A. Immunofluorescence of REH cells staining for DAPI, Sig15, and LAMP2, a marker of the lysosomal compartment. Imaging depicts modest co-localization of Sig15 with LAMP2 in unstimulated REH cells. SIG15 was probed using the 1F7 monoclonal antibody (NextCure). **B.** 3D representative z-stack images of REH cells treated with vehicle control (DMSO) or 81 nM PMA for 24 hours, from which **Fig. 3C** representative images are derived. Cells were stained for Sig15 (green), Golgin-97 (red), and DAPI (blue). PMA stimulation decreases the colocalization of Sig15 and Golgin-97 signal. **C.** Flow cytometry of unpermeabilized REH cells dosed with 81 nM PMA at various timepoints, demonstrating that peak Sig15 localization at the cell surface occurs at approximately 8 hours post-stimulation with a modest, maintained increase at 24 hours (dotted line represents isotype MFI).
