# Supplementary material for: Siglec-15 Promotes Evasion of Adaptive Immunity in B-cell Acute Lymphoblastic Leukemia
Source: Cancer Res Commun. 2023 Jul 17;3(7):1248–59. doi: 10.1158/2767-9764.CRC-23-0056 (PMC10351425; doi:10.1158/2767-9764.CRC-23-0056)
Supplement: Supplemental Figure 4 — Sig15 is required for immune evasion by murine B-ALL. [file crc-23-0056-s04.pdf]

Supplementary Figure 4

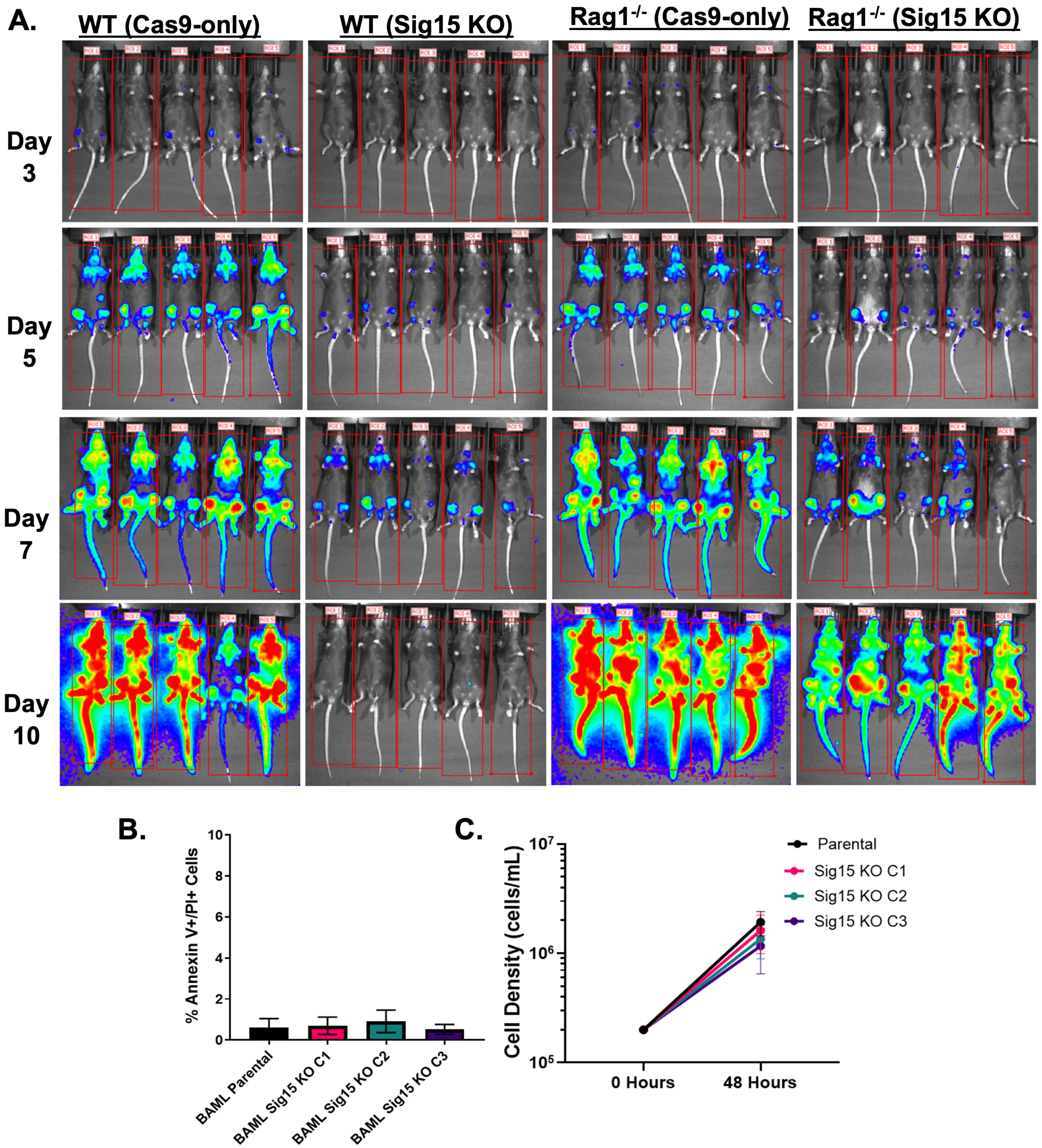

Supplementary Figure 4. Sig15 is required for immune evasion by murine B-ALL. A. IVIS images from the first 14 days of one murine survival study, showing leukemia progression in WT or *Rag1*$^{-/-}$ recipients of Cas9-only or Sig15 KO leukemia as measured by IVIS. **B.** Flow cytometry of three monoclonal knockouts of *Siglec15* in mB-ALL and the parental cell line to assess basal apoptosis levels as measured via Annexin V and Propidium iodide double-positive staining. *Siglec15* knockout did not alter basal apoptosis levels as compared to parental cells. Clone 3 was selected for further analysis in murine experiments. **C.** Cell proliferation of three monoclonal knockouts of *Siglec15* in mB-ALL and the parental cell line as measured by cellometer at 48 hours. *Siglec15* knockout did not alter proliferation of the mB-ALL cells as compared to parental.
