# Supplementary material for: Siglec-15 Promotes Evasion of Adaptive Immunity in B-cell Acute Lymphoblastic Leukemia
Source: Cancer Res Commun. 2023 Jul 17;3(7):1248–59. doi: 10.1158/2767-9764.CRC-23-0056 (PMC10351425; doi:10.1158/2767-9764.CRC-23-0056)
Supplement: Supplemental Figure 5 — Flow cytometry gating schemes. [file crc-23-0056-s05.pdf]

Supplementary Figure 5

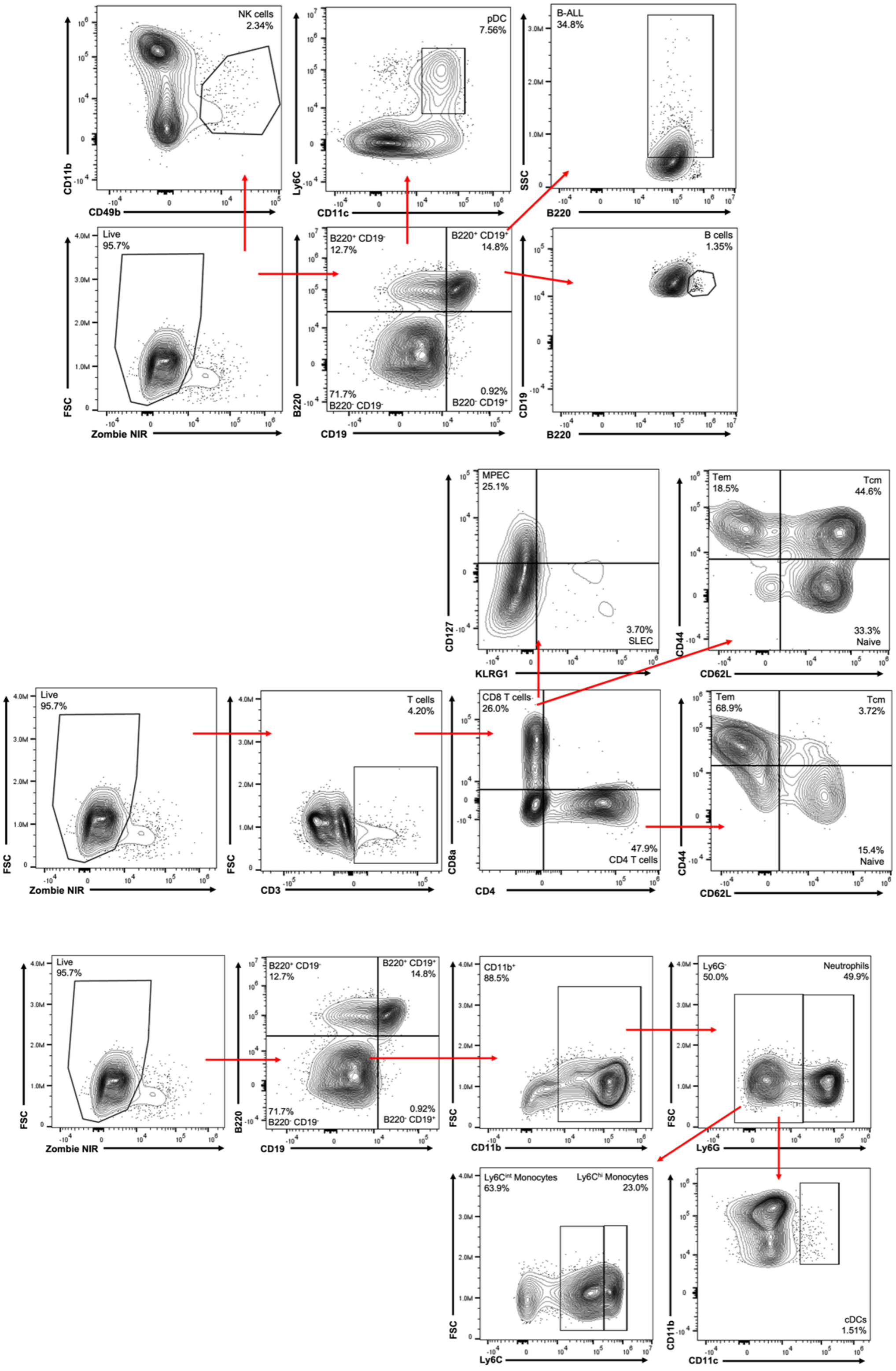

Supplementary Figure 5. Flow cytometry gating schemes for *in* vivo bone marrow immunophenotyping of Sig15 KO in murine B-ALL. Cells are pre-gated on forward/side scatter and singlets prior to the viability gate in Zombie NIR
