# Supplementary material for: Siglec-15 Promotes Evasion of Adaptive Immunity in B-cell Acute Lymphoblastic Leukemia
Source: Cancer Res Commun. 2023 Jul 17;3(7):1248–59. doi: 10.1158/2767-9764.CRC-23-0056 (PMC10351425; doi:10.1158/2767-9764.CRC-23-0056)
Supplement: Supplemental Figure 6 — Sig15 ablation in murine B-ALL promotes an adaptive immune response against leukemia. [file crc-23-0056-s06.pdf]

Supplementary Figure 6

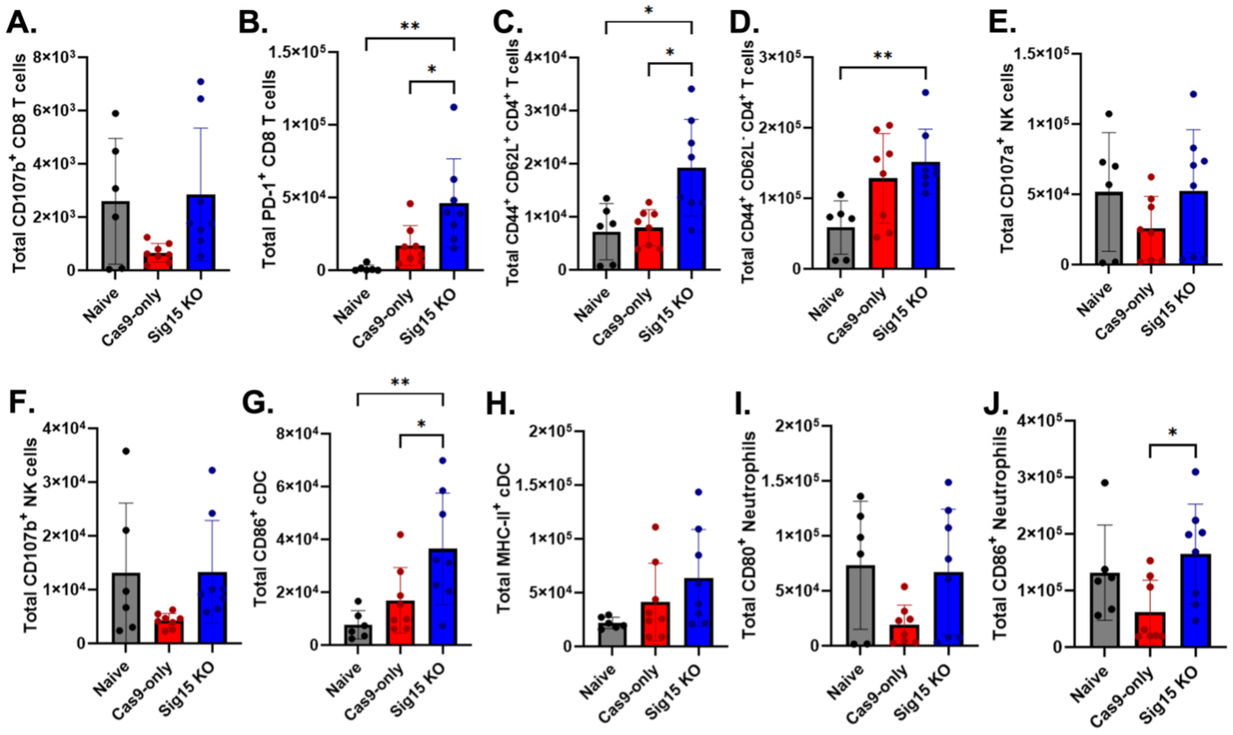

Supplementary Figure 6. Sig15 ablation in murine B-ALL promotes an adaptive immune response against leukemia. A-J. Un-irradiated WT C57BL6 mice were injected via tail vein with 5x10^5 Cas9-only or Sig15 KO cells. Bone marrow was harvested 7 days later for highly dimensional flow cytometry of non-leukemia bone marrow populations. **A.** CD107b as a marker of degranulation was non-significantly higher in Sig15 KO recipients compared to Cas9-only control leukemia. **B.** PD-1 as a marker of CD8$^+$ T cell activation was significantly higher in Sig15 KO recipients. **C-D.** Memory-like early CD4$^+$ Tcm but not Tem populations were significantly higher in Sig15 KO recipients. **E-F.** Degranulation of NK cells in Sig15 KO recipients had a non-significant increase comparable to baseline levels. **G-H.** CD86 costimulatory expression but not MHC-II expression was significantly higher in Sig15 KO recipients. **I-J.** CD86 but not CD80 costimulatory expression on neutrophils was significantly higher in Sig15 KO recipients.
